# Metabolomic approach to evaluating adriamycin pharmacodynamics and resistance in breast cancer cells

**DOI:** 10.1007/s11306-013-0517-x

**Published:** 2013-03-20

**Authors:** Bei Cao, Mengjie Li, Weibin Zha, Qijin Zhao, Rongrong Gu, Linsheng Liu, Jian Shi, Jun Zhou, Fang Zhou, Xiaolan Wu, Zimei Wu, Guangji Wang, Jiye Aa

**Affiliations:** 1 Lab of Metabolomics, Key Laboratory of Drug Metabolism and Pharmacokinetics, State Key Laboratory of Natural Medicines, China Pharmaceutical University, Nanjing, 21009 China; 2 School of Pharmacy, The University of Auckland, Auckland, 1142 New Zealand

**Keywords:** Cellular metabolomics, Adriamycin, Breast cancer MCF-7 cell line, Drug resistance, Reactive oxygen species, Biomarkers

## Abstract

Continuous exposure of breast cancer cells to adriamycin induces high expression of P-gp and multiple drug resistance. However, the biochemical process and the underlying mechanisms for the gradually induced resistance are not clear. To explore the underlying mechanism and evaluate the anti-tumor effect and resistance of adriamycin, the drug-sensitive MCF-7S and the drug-resistant MCF-7Adr breast cancer cells were used and treated with adriamycin, and the intracellular metabolites were profiled using gas chromatography mass spectrometry. Principal components analysis of the data revealed that the two cell lines showed distinctly different metabolic responses to adriamycin. Adriamycin exposure significantly altered metabolic pattern of MCF-7S cells, which gradually became similar to the pattern of MCF-7Adr, indicating that metabolic shifts were involved in adriamycin resistance. Many intracellular metabolites involved in various metabolic pathways were significantly modulated by adriamycin treatment in the drug-sensitive MCF-7S cells, but were much less affected in the drug-resistant MCF-7Adr cells. Adriamycin treatment markedly depressed the biosynthesis of proteins, purines, pyrimidines and glutathione, and glycolysis, while it enhanced glycerol metabolism of MCF-7S cells. The elevated glycerol metabolism and down-regulated glutathione biosynthesis suggested an increased reactive oxygen species (ROS) generation and a weakened ability to balance ROS, respectively. Further studies revealed that adriamycin increased ROS and up-regulated P-gp in MCF-7S cells, which could be reversed by *N*-acetylcysteine treatment. It is suggested that adriamycin resistance is involved in slowed metabolism and aggravated oxidative stress. Assessment of cellular metabolomics and metabolic markers may be used to evaluate anti-tumor effects and to screen for candidate anti-tumor agents.

**Electronic supplementary material:**

The online version of this article (doi:10.1007/s11306-013-0517-x) contains supplementary material, which is available to authorized users.

## Introduction

Adriamycin, a DNA intercalating agent, is an active and popular agent conventionally used for breast cancer management (Gewirtz 1999; Jassem et al. 2009). Multiple mechanisms that are involved in its anticancer activity have been characterized, including direct intercalation between DNA bases, DNA strand breaks, free oxygen radical generation, increased lipid peroxidation, membrane structure alterations(Cummings et al. 1991; Gewirtz 1999), and effects on apoptosis pathways through caspase-3 activation(Gamen et al. 2000) and p21 induction (Ravizza et al. 2004). Unfortunately, continuous adriamycin treatment usually causes chemoresistance, which greatly challenges breast cancer treatment. It has been estimated that a significant number of breast cancer patients (up to 50 %) are not responsive to current chemotherapeutic regimens (O’Driscoll and Clynes 2006). An in vitro study using the popular MCF-7 breast cancer-derived cell line demonstrated that chronic exposure of a sensitive breast cancer cell line (MCF-7S) to adriamycin produces MCF-7Adr, a stable, resistant cell line. To explore the underlying mechanism of adriamycin-induced chemoresistance, genomics and proteomics were recently applied, and the results revealed some candidate genes and proteins that are involved in breast cancer drug resistance. Relative to the sensitive MCF-7S cells, increased epithelial-mesenchymal transition and extracellular matrix composition contributed to adriamycin-resistant MCF-7Adr cell chemoresistance (Iseri et al. 2010, 2011). A proteomic analysis of the chemoresistant and sensitive MCF-7 cells identified proteins that are involved in apoptosis, the cell cycle, glucose metabolism and fatty acid oxidation as contributing to drug resistance (Chuthapisith et al. 2007; Strong et al. 2006).

Although resistance-related genes and proteins were examined, and previous studies on MCF-7Adr and MCF-7S cells suggested that elevated glycolysis, lactate and ATP production, and sulfur-containing amino acids were involved in MCF-7Adr chemoresistance (Broxterman et al. 1989; Lyon et al. 1988; Ryu et al. 2011), little is known about the metabolic patterns and shifts between the sensitive and resistant breast cancer cells (Budczies et al. 2012; Denkert et al. 2012), especially after exposure to chemotherapeutic drugs such as adriamycin. Tumor cells are characterized by reprogrammed metabolic pathways including enhanced anaerobic glycolysis and reduced tricarboxylic acid cycle activity, i.e., the Warburg effect, alternative utilization of glutamine as an energy supply, and they display a role for glycine in rapid cancer cell proliferation (DeBerardinis et al. 2008; Jain et al. 2012; Tomita and Kami 2012). Metabolomics allows for the high-throughput analysis of low molecular mass cellular compounds, which reflects metabolic shifts that are involved in physio-biological processes and may reveal the underlying mechanisms that are involved in these processes. Comparative studies of the differential effects of chemotherapeutic agents on drug-sensitive MCF-7S and drug-resistant MCF-7Adr cells may provide useful information to understand the mechanisms underlying chemoresistance and to assess drug efficacy or resistance. In this study, metabolomics was employed to profile culture media and intracellular metabolites in MCF-7Adr and MCF-7S cells before and after adriamycin exposure. Metabolic patterns and potential markers were identified to evaluate pharmacodynamics and probe for mechanisms underlying adriamycin chemoresistance.

## Materials and methods

### Materials and reagents

Myristic-1,2-^13^C2 acid, 99 atom % ^13^C, the stable isotope-labeled internal standard compound (IS), methoxyamine hydrochloride (purity 98 %), standard alkane solution (C8–C40), and pyridine (≥99.8 % GC) were purchased from Sigma-Aldrich. MSTFA (*N*-methyl-*N*-trimethylsilyl trifluoroacetamide) and 1 % trimethylchlorosilane (TMCS) were obtained from Pierce Chemical Company (Rockford, USA). Methanol and *n*-heptane were HPLC grade and obtained from the Tedia Company (Fairfield, USA) and Merck (Darmstadt, Germany), respectively. Purified water was produced by a Milli-Q system (Millipore, Bedford, USA).

### Cell cultivation and harvest

Human MCF-7 breast cancer cells and the adriamycin-resistant subline (MCF-7Adr) were obtained from the Institute of Hematology and Blood Diseases Hospital (Tianjin, China). These cell lines were grown in RPMI-1640 media supplemented with 10 % (v/v) fetal bovine serum, 100 U/mL penicillin and 0.1 mg/mL streptomycin (Invitrogen, Carlsbad, CA) at 37 °C with 5 % CO_2_. To make the amount of intracellular adriamycin of no significant difference, adriamycin concentrations were examined in MCF-7S and MCF-7Adr cells using the LC–MS/MS assay as previously described (Zhang et al. 2012). Both cell lines were cultured in 6-well plates, grew to nearly 90 % confluent. MCF-7S cells were incubated with 1 μM adriamycin (commercially available in Sigma-Aldrich), while MCF-7Adr cells were incubated with 5 μM adriamycin, respectively, for 6, 12, 18, 24, 36 h (*n* = 6). At each time point, 100 μL of the culture medium in each well was firstly collected, with the remained medium discarded. The adherent cells were washed with cold isotonic saline (0.9 % NaCl, w/v) twice immediately before 300 μL of cold water was added to each well. To quench metabolism, the dishes were stored at −70 °C until extraction. Protein concentrations were assessed to normalize relative metabolite abundance of cell number.

### Sample preparation and GC/TOF–MS analysis

To facilitate the extraction of intracellular metabolites, the cell samples were firstly subjected to three freeze–thaw cycles (freezing at −70 °C for 60 min; thawing at 37 °C for 20 min), and then the adherent cells were flushed with the solution 10 times to ensure the thorough detachment of the cells. For extraction of intracellular metabolites, 900 μL methanol containing 1.5 μg of (^2^C_13_)-myristic acid as an IS was added. The suspension of cell debris in each dish was transferred to an eppendorf tube, vigorously vortexed for 5 min, and then centrifuged at 20,000×*g* for 10 min at 4 °C. For extraction of extracellular metabolites in the culture medium, 100 μL the culture medium were added and extracted with 300 μL methanol containing 0.5 μg of (^2^C_13_)-myristic acid as an IS. The supernatant (300 μL) from both the medium and cell lysate was evaporated to dryness using SPD2010-230 SpeedVac Concentrator (Thermo Savant, Holbrook, USA). 30 μL of methoxyamine in pyridine (10 mg/mL) were added to the dried residue and vigorously vortex-mixed for 2 min. The methoximation reaction was carried out for 16 h at room temperature, followed by trimethylsilylation for 1 h by adding 30 μL of MSTFA with 1 % TMCS as the catalyst. At last, the solution was vortex-mixed again for 30 s after the external standard methyl myristate in heptane (30 μg/mL) was added into each GC vial. The GC/TOF–MS metabolomics analyses were performed as previously described (A et al. 2005; Cao et al. 2011). Briefly, the derivatized sample (0.5 μL) was injected into a 10 m × 0.18 mm ID fused-silica capillary column chemically bonded with 0.18 μm DB-5MS stationary phase (J&W Scientific) in an Agilent 6890 GC system, and the analytes in the eluent were introduced into and detected in a Pegasus III TOFMS (Leco Corp., St. Joseph, MI, USA) as described previously (A et al. 2005; Cao et al. 2011). Mass spectrum was scanned and collected (50–680 *m*/*z*) at a rate of 30 spectra/s after a 170 s solvent delay. Automatic peak detection and mass spectral deconvolution were performed using ChromaTOF™ software (Leco, version 3.25), as reported previously (Jonsson et al. 2005).

### Metabolite identification

Metabolites were identified by comparing the mass spectra and retention indices of the detected compounds with reference standards and those available in the following databases: the National Institute of Standards and Technology (NIST) library 2.0 (2008), Wiley 9 (Wiley–VCH Verlag GmbH & Co. KGaA, Weinheim, Germany), and an in-house mass spectra library database established by the Umeå Plant Science Center, Swedish University of Agricultural Sciences (Umeå, Sweden).

### Intracellular reactive oxygen species (ROS) assay

MCF-7/S and MCF-7/ADR cells were cultured in 12-well plates and treated with 0.2, 1.0, or 5.0 μM adriamycin for 2, 6, 12, or 24 h. After treatment, intracellular ROS was analyzed from the conversion of nonfluorescent DCFH-DA (Beyotime, Jiangsu, China) to its fluorescent derivative. Fluorescence intensity was detected at 535 nm (with 488 nm excitation) in a Synergy-H1 fluorimeter (Bio-Tek Instruments).

Protein concentrations were measured using a BCA protein assay kit (Pierce Chemical, Rockford, IL) according to the manufacturer’s instructions. ROS levels were normalized to protein levels in each sample.

### Western blot

After treatment, cytosolic and membrane proteins were prepared for Western blot analysis as described previously (Zhang et al. 2010). In brief, the cells were scraped and lysed. Protein concentrations were measured using a BCA protein assay kit (Pierce Chemical, Rockford, IL) according to the manufacturer’s instructions. The samples were reconstituted in SDS-polyacrylamide gel electrophoresis sample loading buffer, and proteins were denatured by boiling for 5 min. Equal protein amounts were separated on an 8 % SDS–polyacrylamide gel and transferred onto a PVDF membrane (Millipore Corporation).

After blotting, the membrane was blocked with 10 % BSA in TBS/T for 1 h at 37 °C, The immunoblots were incubated with human anti-P-gp antibodies (1:800, clone 3C3.2, Millipore, USA) for 48 h or anti-β-actin antibodies (1:800, Bioworld, USA) for 24 h at 4 °C. After washing with TBST, the membranes were incubated with HRP-conjugated secondary antibodies (KeyGen, Nanjing, China) for 1 h. The signal was visualized by enhanced chemiluminescence (ECL, Millipore, MA, USA).

### RNA extraction and quantitative PCR analysis

Total RNA was isolated from cultured cells using Trizol reagent (Invitrogen). First-strand complementary DNA synthesis with a reverse transcription polymerase chain reaction (RT-PCR) kit was performed with 1 μg of RNA (TaKaRa Bio Inc.). RT-qPCR was performed in a CFX96 real-time RT-PCR detection system with a C1000 thermal cycler (Bio-Rad, USA). The reactions were performed in a 15 μL volume containing 7.5 μL of 2 × SYBR Premix Ex Taq (TaKaRa Bio Inc.), 2 μL of diluted cDNA, and 1 μM primers. The primers used in this study were as follows: β-actin forward primer: GCGTGACATTAAGGAGAAG, reverse primer: GAAGGAAGGCTGGAAGAG; P-gp forward primer: GCTGGGAAGATCGCTACTGA, reverse primer: GGTACCTGCAAACTCTGAGCA; GPDH forward primer: GGTAGACAAGTTTCCCTT, reverse primer: ATATGTTCTGGATGATTCTG. Thermal cycling conditions included 95 °C initial denaturation for 5 min. followed by 40 cycles of denaturation (10 s at 95 °C), annealing (15 s at 56–61 °C) and extension (15 s at 72 °C with a single fluorescence measurement), a melt curve program (60–95 °C with a 0.11 °C/s heat increase and continuous fluorescence measurement) and a cooling step to 40 °C. Relative mRNA levels were calculated by the comparative threshold cycle method using β-actin as the internal control.

### Multivariate data analysis

The relative quantitative peak areas of each detected peak were normalized to myristic-1,2-^13^C_2_ acid, the stable isotope IS, before a multivariate statistical analysis using SIMCA-P 11 software (Umetrics, Umeå, Sweden). Heatmaps were generated with R-Project, which is available online at: http://www.r-project.org/ (Vanderbilt University, Nashville Tennessee, USA). Here, a principal component analysis (PCA) and a partial least squares projection to latent structures and discriminant analysis (PLS–DA) were employed to process the acquired GC/TOF–MS data. Samples from the same groups were grouped together for PLS–DA modeling. The PCA and PLS–DA results were displayed as scores plots to visualize sample clustering and to indicate sample similarity. The model goodness of fit was evaluated using three quantitative parameters; i.e., R^2^X is the explained variation in X, R^2^Y is the explained variation in Y, and Q^2^Y is the predicted variation in Y. Cross-validation with seven groups was used throughout to determine the principal component number (Eriksson et al. 2001; Trygg et al. 2007; Wold 1978), which was determined once the Q^2^Y value decreased continuously. Permutation tests were performed with 100 iterations to validate the model. Discriminatory metabolites between the treated and the un-treated control at each time point were first screened in the column plot and then validated using a one-way analysis of variance with a significance level of 0.05 for three time points (e.g., 6, 12, 24 h). The impact of adriamycin on metabolic pathways was evaluated based on a tool for metabolomic data analysis, which is available online (http://www.metaboanalyst.ca/MetaboAnalyst/faces/Home.jsp) (Xia et al. 2009). The Pathway Analysis module combines results from powerful pathway enrichment analysis with the pathway topology analysis to help researchers identify the most relevant pathways involved in the conditions under study. By uploading the discriminatory compounds that were significantly influenced by adriamycin treatment, the built-in Homo sapiens (human) pathway library for pathway analysis and hypergeometric test for over-representation analysis were employed. A results report was then presented graphically as well as in a detailed table. Potential drug efficacy and/or resistance biomarkers were identified based on the identified metabolic pathways and the statistics.

### Statistics

Values are presented as the mean ± SD. Differences between data sets were analyzed by a one-way ANOVA, and *p* < 0.05 was considered to be statistically significant.

## Results

### Adriamycin distinctly perturbed metabolic patterns and intracellular metabolites in MCF-7S cells

**Fig. 1:**
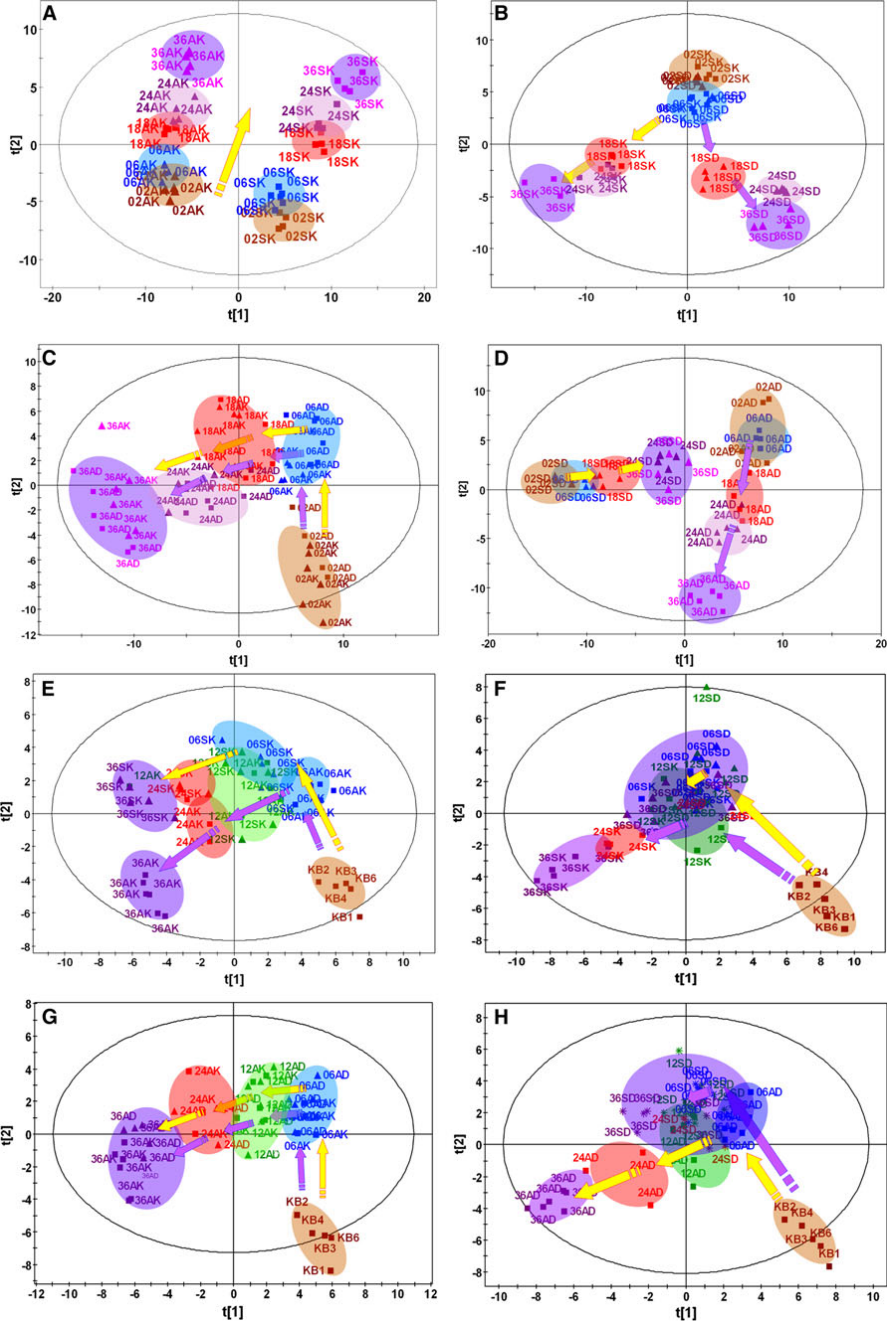
Metabolic patterns of intracellular metabolites and culture media metabolites in adriamycin-treated or untreated MCF-7S and MCF-7Adr cells. **a** Time-dependent metabolic patterns of metabolites in MCF-7S and MCF-7Adr cell lines without adriamycin. **b** Time-dependent metabolic patterns of metabolites in MCF-7S cells with or without adriamycin. **c** Time-dependent metabolic patterns of metabolites in MCF-7Adr cells with or without adriamycin. **d** Time-dependent metabolic patterns of metabolites in MCF-7S and MCF-7Adr cell lines with adriamycin. **e** Time-dependent metabolic patterns of culture media metabolites in untreated MCF-7S and MCF-7Adr cell lines. **f** Time-dependent metabolic patterns of metabolites in untreated or adriamycin-treated MCF-7S cell culture media. **g** Time-dependent metabolic patterns of metabolites in untreated or adriamycin-treated MCF-7Adr cell culture media. **h** Time-dependent metabolic patterns of metabolites in adriamycin-treated MCF-7S and MCF-7Adr cell culture media. *SK* sensitive MCF-7S cells, *AK* resistant MCF-7Adr cells, *SD* adriamycin-treated MCF-7S cells, *AD* adriamycin-treated MCF-7Adr cells, *KB* original culture media. The numbers 02, 06, 18, 24, and 36 indicated that the cells were treated with adriamycin for 2, 6, 18, 24, or 36 h, respectively. Parameters of each model were summarized in Electronic Supplementary Material, Table S-6

To evaluate metabolic effect of adriamycin on MCF-7S and MCF-7A, comparable concentration of intracellular adriamycin is of crucial importance. Measurement of intracellular adriamycin showed that there was not significant difference between MCF-7S (1.84 ± 0.11 nmol/mg protein) and MCF-7A (1.75 ± 0.12 nmol/mg protein, *n* = 4) when MCF-7S and MCF-7A were exposed to adriamycin at 1 μM and 5 μM respectively for 12 h. A PCA based on cellular metabolites demonstrated that the two cell lines clustered closely within each group and separately from each other, indicating different metabolic patterns between the sensitive MCF-7S and the resistant MCF-7Adr cells (Fig. 1a). Both the sensitive MCF-7S and resistant MCF-7Adr cells moved similarly from the bottom up in the scores plot as the cells were cultivated for 2, 6, 18, 24 and 36 h (Fig. 1a). Adriamycin exposure markedly perturbed the MCF-7S metabolic pattern and re-directed cellular movement the other way (Fig. 1b), indicating a distinct modulation of the sensitive MCF-7S cell metabolism.

**Fig. 2:**
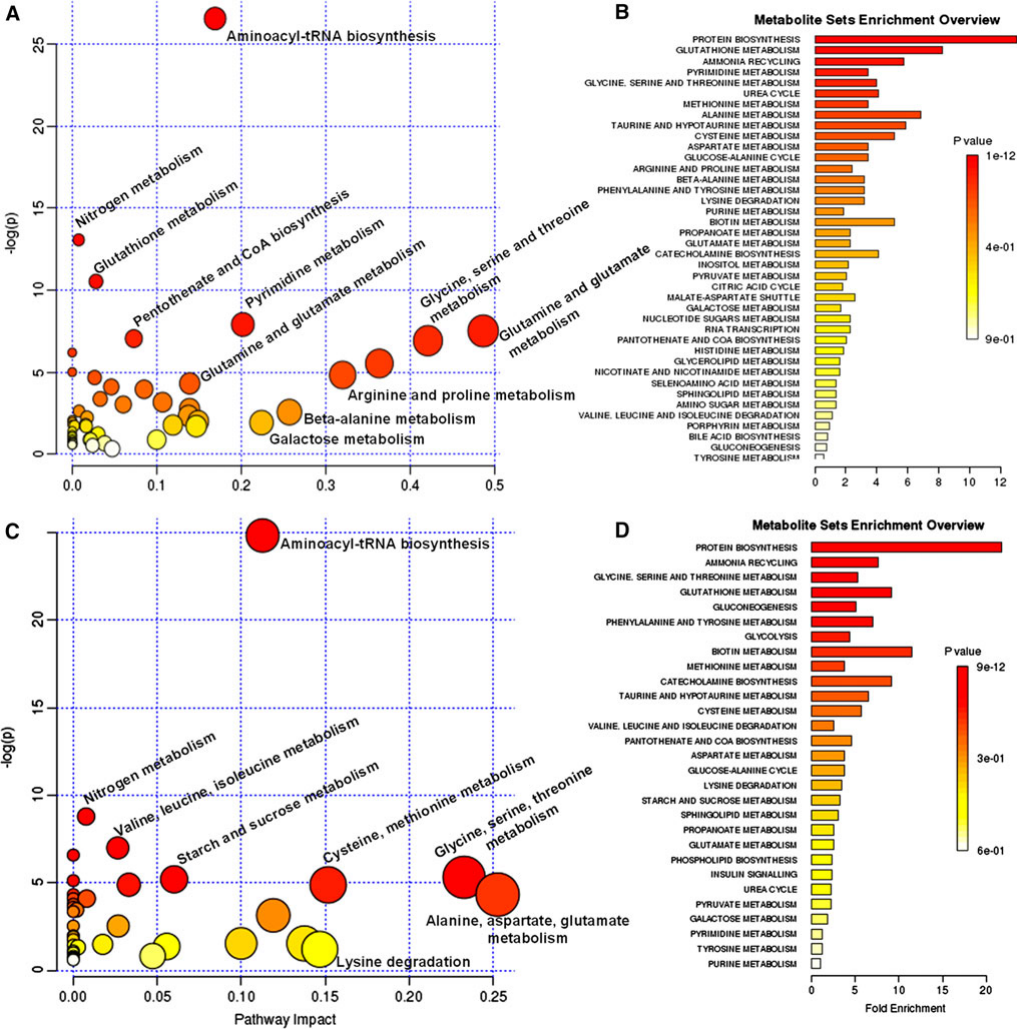
The impact of adriamycin on MCF-7S cell metabolic pathways. **a** Intracellular metabolite-based metabolic pathway analysis of MCF-7S cells. **b** Overview of metabolites that were enriched in MCF-7S cells based on MCF-7S cell intracellular metabolites. **c** Metabolic pathway analysis of MCF-7S cells based on the MCF-7S cell culture media metabolites. **d** Overview of metabolites that were enriched in MCF-7S cells based on the MCF-7S cell culture media metabolites

**Fig. 3:**
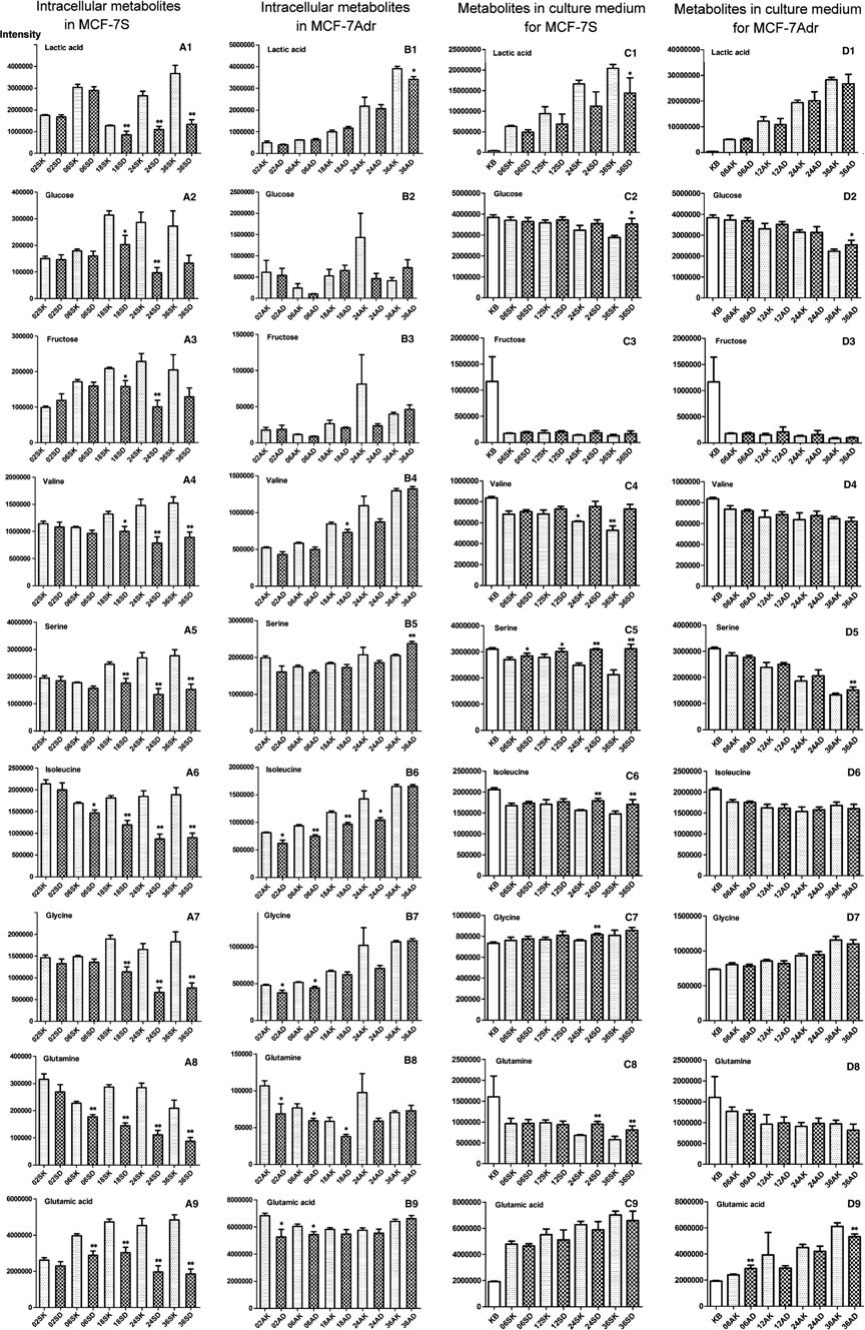
Metabolic effect of adriamycin on the relative abundance of intracellular and culture media metabolites. *KB* original culture media, *SK* sensitive MCF-7S cells, *AK* resistant MCF-7Adr cells, *SD* adriamycin-treated MCF-7S cells, *AD* adriamycin-treated MCF-7Adr cells treated with adriamycin, *KB* original culture media. The numbers 02, 06, 12, 18, 24, and 36 indicated that the cells were treated with adriamycin for 2, 6, 12, 18, 24, or 36 h, respectively. **A1**–**A9**, **B1**–**B9**, **C1**–**C9** and **D1**–**D9** represents intracellular metabolites in MCF-7S cells, intracellular metabolites in MCF-7Adr cells, extracellular substance in MCF-7S cells, extracellular substance in MCF-7Adr cells, Data are presented as mean ± SE *p ≤ 0.05; **p ≤ 0.01 versus control

Statistics and metabolite identification revealed that many metabolites were severely perturbed when the sensitive MCF-7S cells were exposed to adriamycin (Online Resource 1, Table S-1 and Online Resource 2, Figure S-1). A metabolic pathway analysis demonstrated that adriamycin affected amino acid turnover or protein biosynthesis (glutamine, glycine, isoleucine, valine, proline, tyrosine, lysine, phenylalanine, taurine, threonine, serine, tryptophan, aminomalonic acid), glutathione metabolism (cysteine, glutamic acid, cystathionine, glycine, methionine, Cys–Gly), pyrimidine metabolism (thymine, uracil, urindine, beta-alanine, 5′-uridine monophosphate), purine metabolism (xanthine, adenine, inosine), glycolysis (lactic acid, glucose, fructose, inositol), glycerol metabolism (glycerol, glyceric acid, glycerol-3-phosphate), the tricarboxylic acid cycle (citric acid, malic acid) and the glucose-alanine cycle (glucose, alanine) (Fig. 2a, b, Table S-1, Online Resource 2, Table S-2). In MCF-7S cells, adriamycin significantly perturbed the levels of the metabolites that are involved in the above pathways compared with the untreated control, and some data are shown in Fig. 3.

### Adriamycin had less effects on metabolic patterns and intracellular metabolites in MCF-7Adr cells

In contrast to MCF-7S cells, adriamycin exposure did not have obvious effects on the metabolic pattern of MCF-7Adr cells. In fact, when treated with adriamycin, the MCF-7Adr cells were similar to the resistant controls, as seen in Fig. 1c. The marginally changed metabolic pattern of the adriamycin-exposed MCF-7Adr cells indicated that adriamycin had little effect on modulating MCF-7Adr metabolism, reflecting the adriamycin resistance of MCF-7Adr cells. Moreover, after adriamycin exposure, the sensitive MCF-7S cells moved shorter distance and more slowly (Aa et al. 2011) than MCF-7Adr (Fig. 1d), indicating that adriamycin inhibited metabolism/metabolites more in the MCF-7S cells than in the MCF-7Adr cells and suggesting that MCF-7Adr cells were resistant to adriamycin. Interestingly, exposing the sensitive MCF-7S cells to adriamycin caused the metabolites to shift more towards that of MCF-7Adr (Fig. 1d, Online Resource 2, Figure S-2). This result suggests that adriamycin treatment reprogrammed the metabolic pattern of MCF-7S cells to be similar to MCF-7Adr cells.

Statistics and metabolite identification revealed that some metabolites (valine, isoleucine, proline) were significantly perturbed in adriamycin-exposed MCF-7Adr cells (Online Resource 1, Table S-3). Adriamycin marginally affected glycine, serine, threonine, cysteine, phenylalanine, taurine, tyrosine, Cys–Gly, adenine, aminomalonic acid, malic acid, asparagine and glutamine levels relative to the untreated MCF-7Adr control, and these levels were much less affected than in the adriamycin-exposed MCF-7S cells (some data are shown in Fig. 3).

### Adriamycin distinctly perturbed metabolic patterns and metabolites in the MCF-7S culture media

Metabolic patterns of the sensitive MCF-7S cells were evaluated based on culture media metabolites. It was demonstrated that the metabolome changed markedly from the original culture within the first 6 h after treatment, as seen in Fig. 1e, followed by a slower modifications. The continuous movement of the score plots reflected the gradual influx and efflux of substances between the culture media and the cells (Fig. 1e); i.e., nutritious substances such as amino acids (serine, valine, isoleucine, glutamine, cysteine) and saccharides (glucose, fructose) were consumed, and there was a distinct accumulation of glutamic acid and metabolic products such as lactic acid (Fig. 3). Except for the gap within the first 6 h, there was no obvious movement of the sensitive MCF-7S cells from 6 to 12, 24 or even 36 h after adriamycin treatment (Fig. 1f). These data indicated that adriamycin had a substantial effect to slow down MCF-7S cell metabolism and, hence, the exchange of substances between the cells and the culture media. Consistent with the intracellular metabolites in MCF-7S cells, adriamycin significantly increased valine, isoleucine, serine, threonine, pyroglutamic acid, glucose, glutamine, phenylalanine, tyrosine, and ribitol levels in the culture media relative to the control (Fig. 3; Table S-1, Online Resource 2, Table S-4 and Figure S-3), indicating less consumption of these metabolites in adriamycin-treated cells and an impact on the involved metabolic pathways (Fig. 2c, d). However, relative to the intracellular metabolites, the discriminatory metabolite number and their fold-changes were less perturbed by adriamycin treatment. It was further suggested that the metabolic profile of the culture media metabolites can be used to assess the pharmacodynamics of anti-tumor agents.

### Adriamycin had little effect on the metabolic pattern and culture media metabolites in MCF-7Adr cells

Based on the culture media metabolites, adriamycin exposure had little effect on the metabolic patterns of MCF-7Adr cells. Unlike the response of MCF-7S when exposed to adriamycin, the MCF-7Adr cells moved similar to the resistant controls (Fig. 1g, h). Few metabolites (serine, isoleucine, glutamic acid) were significantly affected after long-term adriamycin exposure (Fig. 3). The MCF-7Adr cell metabolic patterns were marginally changed after adriamycin exposure, indicating that adriamycin only slightly modulated MCF-7Adr metabolism. The differential effect of adriamycin on the MCF-7S and MCF-7Adr cell metabolic patterns strongly enforces the potential for metabolomics in assessing anti-tumor agent resistance.

### Potential biomarkers indicating drug efficacy and adriamycin resistance

**Fig. 4:**
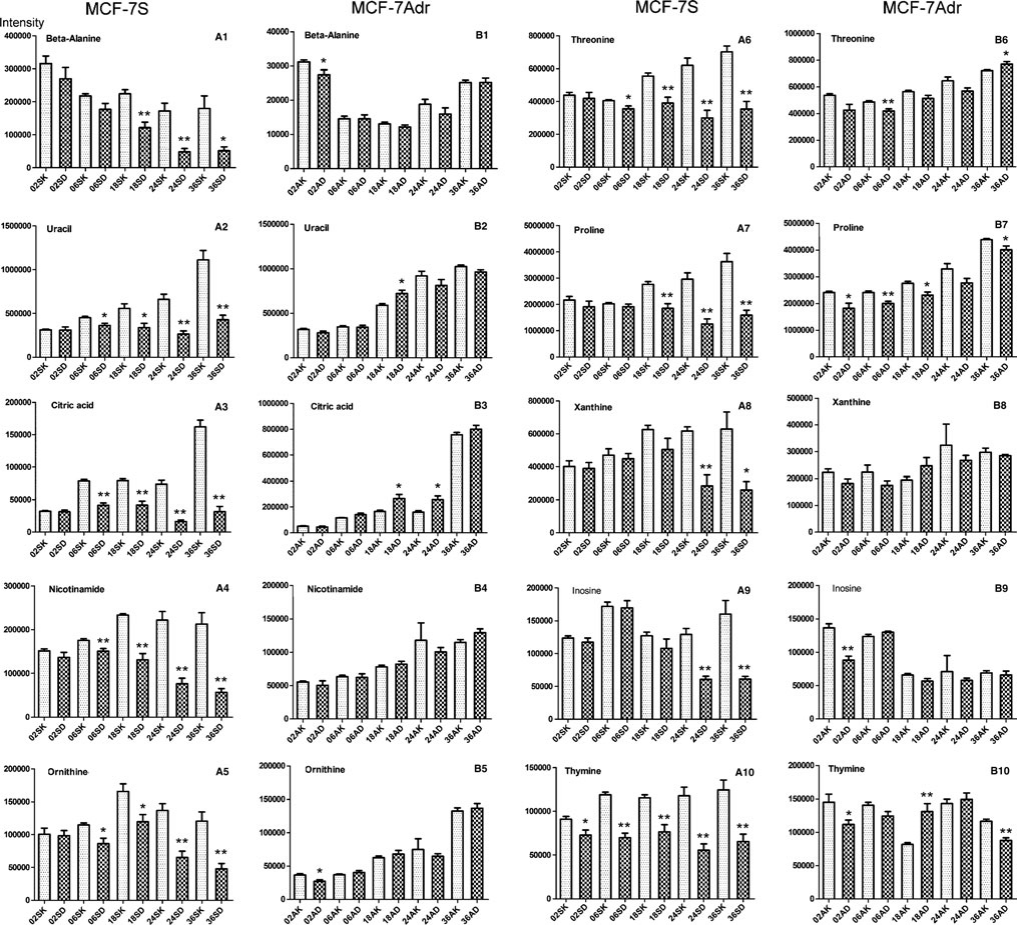
The effect of adriamycin on typical metabolite levels in the sensitive MCF-7S and resistant MCF-7Adr cells. *SK* sensitive MCF-7S cells, *AK* resistant MCF-7Adr cells, *SD* adriamycin-treated MCF-7S cells, *AD* adriamycin-treated MCF-7Adr cells. The numbers 02, 06, 18, 24, and 36 indicated that the cells were treated with adriamycin for 2, 6, 18, 24, or 36 h, respectively. **A1**–**A10** and **B1**–**B10**, represents intracellular metabolites in MCF-7S cells and MCF-7Adr cells respectively. Data are presented as mean ± SE *p ≤ 0.05; **p ≤ 0.01 versus control

Two metabolite groups were affected by adriamycin treatment. First, many intracellular metabolites (valine, isoleucine, proline, glycine, serine, threonine, inosine, thymine, cysteine, ornithine-3TMS, phenylalanine, taurine, aminomalonic acid, malic acid, asparagine, glutamine, adenine, tyrosine, Cys–Gly) responded less to adriamycin in MCF-7Adr cells than in MCF-7S cells (Figs. 3, 4). These metabolites responded similarly in the two cell lines and were therefore indicated as quantitative drug resistance markers. Second, some intracellular metabolites displayed differential responses to adriamycin in MCF-7S and MCF-7Adr cells. In MCF-7S cells, adriamycin significantly decreased beta-alanine, lactic acid, uracil, nicotinamide, glutamic acid, and ornithine-4TMS levels (Fig. 4). However, these parameters were not perturbed in MCF-7Adr cells and were indicated as markers of adriamycin sensitivity and resistance. Interestingly, adriamycin reduced intracellular citric acid levels in MCF-7S cells but had the opposite effect in MCF-7Adr cells (Fig. 4). Thus, citric acid was suggested as a unique marker to represent the antitumor agent sensitivity and resistance.

In the culture media, lactic acid was a useful marker to indicate MCF-7S and MCF-7Adr cell sensitivity or resistance to adriamycin. There was a distinct decline of lactic acid levels in the culture media of MCF-7S cells after adriamycin exposure, while lactic acid levels were not affected in the MCF-7Adr cells (Fig. 3).

### Adriamycin and *N*-acetylcysteine-mediated effects on ROS and P-gp expression

**Fig. 5:**
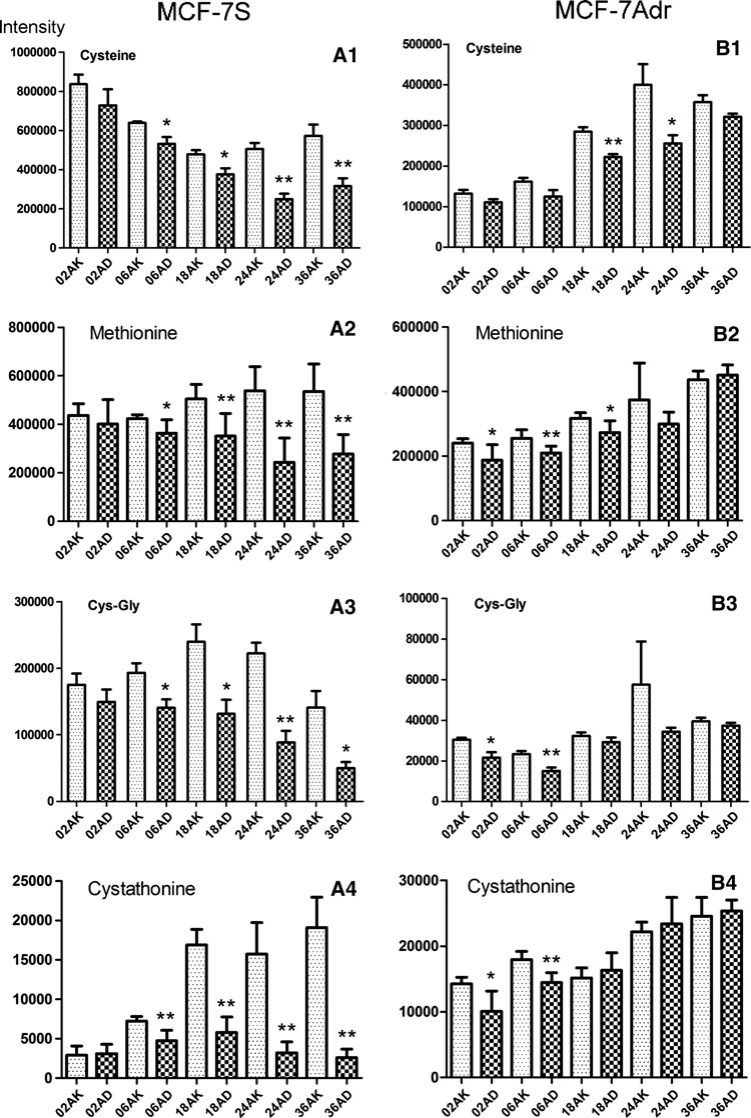
The effect of adriamycin on intracellular glutathione biosynthesis metabolite levels. *SK* sensitive MCF-7S cells, *AK* resistant MCF-7Adr cells, *SD* adriamycin-treated MCF-7S cells, *AD* adriamycin-treated MCF-7Adr cells. The numbers 02, 06, 18, 24, and 36 indicated that the cells were treated with adriamycin for 2, 6, 18, 24, or 36 h, respectively. **A1**–**A4** and **B1**–**B4**, represents intracellular metabolites in MCF-7S cells and MCF-7Adr cells respectively. Data are presented as mean ± SE *p ≤ 0.05; **p ≤ 0.01 versus control

**Fig. 6:**
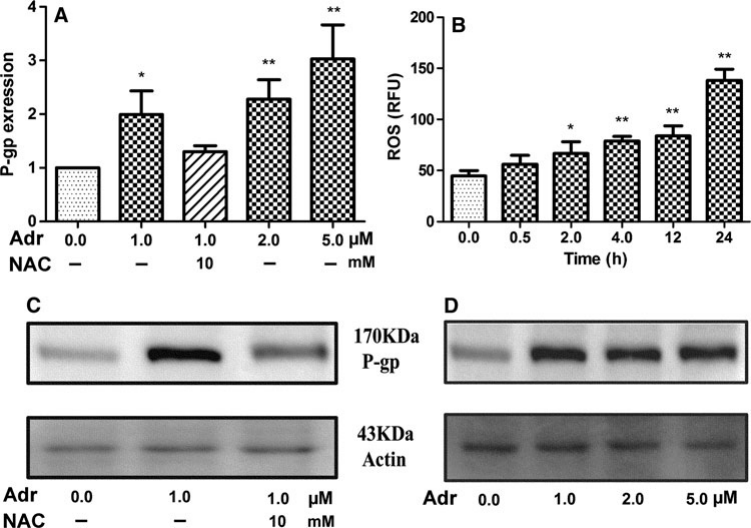
Adriamycin-induced P-gp expression and ROS production in MCF-7S cell line. **a** The effect of adriamycin and *N*-acetylcysteine treatment on P-gp mRNA expression (24 h), **b** time-dependent effect of adriamycin (1 μM) on ROS, **c** the effect of adriamycin and *N*-acetylcysteine treatment on P-gp expression (24 h), **d** concentration-dependent effect of adriamycin on P-gp expression (24 h). Data are presented as mean ± SD. *p ≤ 0.05; **p ≤ 0.01 versus control

Treatment of the MCF-7S cells with adriamycin significantly depressed glutathione synthesis (Figs. 3, 5) and up-regulated glycerol metabolism (Online Resource 2, Figure S-4). Short-term (6–36 h) adriamycin treatment reduced glycerol levels but increased glycerol-3-phosphate levels, while long-term adriamycin treatment (as assessed in MCF-7Adr vs. MCF-7S cells that were made after months of low-level adriamycin treatment) significantly increased glyceric acid, glycerol-3-phosphate, and dihydroxyacetone phosphate (DHAP) levels (Figure S-4), which are involved in ROS generation. Additionally, α-glycerol phosphate dehydrogenase (GDPH), which is important for glycerol-3-phosphate metabolism into DHAP, was significantly elevated in MCF-7S cells after adriamycin exposure (Online Resource 2, Figure S-5). This up-regulated glycerol metabolism suggested that more ROS were generated, while the reduced glutathione synthesis indicated a weakened ability of the cells to balance the elevated ROS. To study the correlation among adriamycin, ROS and P-gp expression, ROS and P-gp expression were examined after adriamycin exposure. The data demonstrated that adriamycin treatment increased ROS levels and up-regulated P-gp expression, which was reversed by *N*-acetylcysteine treatment (Fig. 6).

## Discussion

To the best of our knowledge, this is the first study to evaluate drug efficacy and resistance based on in vitro metabolomic data from cancer cells, although the potential application of metabolomics to assess drug efficacy and safety was proposed 5 years ago(Keun and Athersuch 2007). In the current study, adriamycin-induced metabolic patterns ion MCF-7S and MCF-7Adr cells were characterized. Based on the data obtained from intracellular and culture media metabolites, the two cell lines demonstrated different metabolic patterns after adriamycin treatment. Adriamycin treatment markedly perturbed the metabolic pattern of intracellular metabolites in MCF-7S cells (Fig. 1b), but metabolic patterns of culture media metabolites were not significantly affected after adriamycin treatment for 6, 12, 24 or 36 h (Fig. 1f). These results suggested that adriamycin efficiently inhibited or slowed down cellular metabolism and proliferation to produce its anti-tumor effect. Conversely, adriamycin treatment perturbed the metabolic pattern of the MCF-7Adr cells much less based on the intracellular (Fig. 1c) and cell culture media metabolites (Fig. 1g) compared with the non-treated MCF-7Adr cells. These results suggested that adriamycin inhibited MCF-7Adr cell metabolism and proliferation to a lesser extent, thus indicating chemoresistance. It has been suggested that cellular metabolomics may be used to assess antitumor agent pharmacodynamics and potential drug resistance on cell lines.

Breast cancer chemotherapeutic resistance has drawn much attention in recent years. A few studies documented the relationship between chemotherapeutic resistance and metabolic profiles in other cancer cell lines (Klawitter et al. 2009; Serkova and Boros 2005) and in cancer patients (A et al. 2010). The biochemical effects of adriamycin in animal model was studied (Park et al. 2009), potential metabolomic biomarkers in urine for evaluation of adriamycin efficacy were identified (Kim et al. 2012), and targeted profiling of modified nucleosides was also used to characterize the metabolic signature of the breast cancer cell line MCF-7/S and compared it to the human mammary epithelial cells MCF-10A (Bullinger et al. 2007). However, there are no comparative pharmaco-metabolomics reports using the couple of breast cancer cell lines, i.e., the sensitive MCF-7S and the resistant MCF-7A cells. Our study revealed that the metabolic pattern of the sensitive MCF-7S cells was modulated and eventually became similar to that of the resistant MCF-7Adr cells (Figure S-2), suggesting that this metabolic shift in MCF-7S cells was deeply involved in adriamycin resistance. In the current study, we identified that adriamycin inhibited purine, pyrimidine, and protein biosynthesis in the sensitive MCF-7S cells more strongly than in the resistant MCF-Adr cells, which may be involved in inhibiting cell proliferation, inducing apoptosis(Kim et al. 1998; Kondo et al. 2000; Quemeneur et al. 2003), and inducing methotrexate resistance in osteosarcoma (Cole et al. 2002). The adriamycin-induced down-regulation of purine, pyrimidine and protein synthesis not only explained the small effect of adriamycin on resistant MCF-7Adr cells but might also contributes to adriamycin resistance. Furthermore, adriamycin significantly decreased glutathione biosynthesis in the sensitive MCF-7S cells (Fig. 5) and simultaneously increased glycerol metabolism (Figure S-4). This elevated glycerol metabolism suggested enhanced ROS generation, while the down-regulated glutathione biosynthesis indicated a weakened ability to balance ROS after adriamycin exposure. It was suggested that oxidative stress was involved in adriamycin resistance in MCF-7 cells. Further studies revealed that adriamycin significantly increased ROS and up-regulated P-gp levels in MCF-7S cells, which was efficiently reversed by NAC treatment. These data suggested that adriamycin resistance was involved in balancing oxidative stress and the metabolic shifts mentioned above.

Consistent with a previous report, the discriminatory metabolites that were elucidated in this study, such as increased lipids, decreased glutamine, glutamate, taurine and glutathione levels, were also found as doxorubicin/adriamycin-induced biomarkers in HL60 promyelocytic leukemia cells (Rainaldi et al. 2008). Additionally, our results correlated with a recent study on breast cancer patients in terms of the regulated metabolic pathways and markers, which demonstrated that the breast cancer tissue was characterized by perturbed protein, purine, and pyrimidine biosynthesis; glutamic acid, arginine + proline, alanine + aspartate, glycine + serine + threonine metabolism, and TCA cycle (Budczies et al. 2012). Our data demonstrated that adriamycin affected most of these metabolic pathways in the sensitive MCF-7S cells, indicating that these may be underlying mechanisms involved in the antitumor effect of adriamycin. Moreover, taurine, hypoxanthine, aminomalonic acid, glutamic acid, malic acid, and pyroglutamic acid, which were metabolic markers that were identified in the clinical study, were also identified as potential markers for assessing adriamycin efficacy in the current study, although the utility of these and our markers as a measurement of sensitivity or resistance need to be further studied. It is worth mentioning that in contrast to the blank control, serine in the medium showed a significant increase after adriamycin exposure in sensitive cells, which indicated that cancer cells rapidly depleted exogenous serine while adriamycin inhibited the utilization of serine and induce serine starvation in cells. This change could be observed shortly after MCF-7S cells were treated with drugs for 6 h. Consistently, a recent study showed that serine depletion may result in oxidative stress, reduced viability and severely impaired proliferation of cells (Maddocks et al. 2013). It was suggested that serine starvation is involved in the effect of adriamycin, and extracellular serine in the medium could be a potential marker to indicate the sensitivity or resistance of adriamycin.

Based on the metabolomic data and multivariate statistical analysis, both the scores plots and the metabolic markers clearly revealed strong adriamycin-induced effects on the sensitive MCF-7S cells and quantitatively weak adriamycin-induced effects on the resistant MCF-7Adr cells. According to the relative distance value in the scores plot (Aa et al. 2011), the close clustering of the MCF-7S (Fig. 1f) after adriamycin treatment for 6, 18, 24, or 36 h suggested strong metabolic inhibition, whereas the distant scattering of the MCF-7Adr cells (Fig. 1c, g) after adriamycin treatment for 6, 12, 18, 24, or 36 h suggested weak effects. Conversely, the distance of the treated samples compared with the non-treated cells is also a quantitative indicator for assessing pharmacodynamics. Generally, lower distance values indicate less impact on cell metabolism and poor effectiveness/resistance, while higher values indicate stronger cell metabolism inhibition and/or stronger drug effectiveness relative to the non-treated controls (Fig. 1c, f, g; Online Resource 2, Table S-5). Furthermore, MCF-7Adr cell intracellular and culture media metabolites were less perturbed by adriamycin than were MCF-7S cells. Adriamycin treatment modulated the levels of many intracellular amino acids, lactic acid, beta-alanine, citric acid, uracil, and nicotinamide as well as cell culture media amino acids and lactic acid in the sensitive MCF-7S cells, but these compounds were less affected or were unaffected in the resistant MCF-7Adr cells. These metabolites were indicated as potential markers of adriamycin resistance. Quantitative measurement of these metabolites can potentially assess drug efficacy, and it has been suggested that cellular metabolomics and metabolic markers can assess anti-tumor effects and aid in quantitative screening of candidate anti-tumor agents.

## Conclusions

Adriamycin treatment slowed down several metabolic pathways, such as protein, purine, pyrimidine, and glutathione biosynthesis and glycolysis, yet up-regulated glycerol metabolism. It was suggested that this slowed metabolism and aggravated oxidative stress were involved in adriamycin resistance. Cellular metabolomics and quantitative metabolic markers measurement may potentially be used to evaluate anti-tumor effects and screen candidate anti-tumor agents.

## Electronic supplementary material

Below is the link to the electronic supplementary material.

Supplementary material 1 (XLSX 47 kb)

Supplementary material 2 (DOC 3598 kb)
