# Supplementary material for: Metabolomic approach to evaluating adriamycin pharmacodynamics and resistance in breast cancer cells
Source: Metabolomics. 2013 Mar 20;9(5):960–73. doi: 10.1007/s11306-013-0517-x (PMC3769585; doi:10.1007/s11306-013-0517-x)
Supplement: Supplementary file 2 — Supplementary material 2 (DOC 3598 kb) [file 11306_2013_517_MOESM2_ESM.doc]

**Supplementary materials for manuscript**

Metabonomic approach to evaluating pharmacodynamics and resistance of adriamycin in breast cancer cells

**Check list**

**Table S-1** The effect of adriamycin on metabolites in MCF-7S cells

**Table S-2** Metabolic pathway analysis based on the intracellular metabolites in MCF-7S treated with adriamycin

**Table S-3** The effect of adriamycin on metabolites in MCF-7Adr cells

**Table S-4** Metabolic pathway analysis based on the metabolites in culture media for MCF-7S treated with adriamycin

**Table S-5** Distance moved by each group relative to the non-treated control after exposure to adriamycin based on the PLS-DA models *

**Table S-6** Parameters of the models in Figure 1

**Figure S-1** Heatmap of the intracellular metabolites for MCF-7S cells in response to adriamycin

**Figure S-2** Metabolic shift of MCF-7S cell intracellular metabolites after adriamycin exposure. Adriamycin exposure of the sensitive MCF-7S cells approached the metabolite profile of the MCF-7Adr cells, suggesting that adriamycin reprogrammed the MCF-7S cell metabolic pattern to be similar to MCF-7Adr cells.

A, A PLS-DA model based on intracellular data from resistant MCF-7Adr and sensitive MCF-7S cells that were adriamycin treated or untreated; B, A PLS-DA model based on intracellular data from adriamycin-treated resistant MCF-7Adr cells and sensitive MCF-7S cells.

SK, sensitive MCF-7S cells; AK, resistant MCF-7Adr cells; SD, adriamycin-treated MCF-7S cells; AD, adriamycin-treated MCF-7Adr cells. The numbers 02, 06, 12, 18, 24, and 36 indicated that the cells were treated with adriamycin for 2, 6, 12, 18, 24, or 36 hours, respectively.

**Figure S-3** Heatmap of the metabolites in the culture media for MCF-7S cells in response to adriamycin

**Figure S-4** The effect of adriamycin on intracellular glycerol metabolism metabolites. SK, sensitive MCF-7S cells; AK, resistant MCF-7Adr cells; SD, adriamycin-treated MCF-7S cells; AD, adriamycin-treated MCF-7Adr cells. The numbers 02, 06, 18, 24, and 36 indicate that the cells were treated with Adriamycin for 2, 6, 18, 24, or 36 hours, respectively.

**Figure S-5** mRNA level of GDPH in MCF-7S when exposed to adriamycin for 18 h

**Table S-2** Metabolic pathway analysis based on the intracellular metabolites in MCF-7S treated with adriamycin


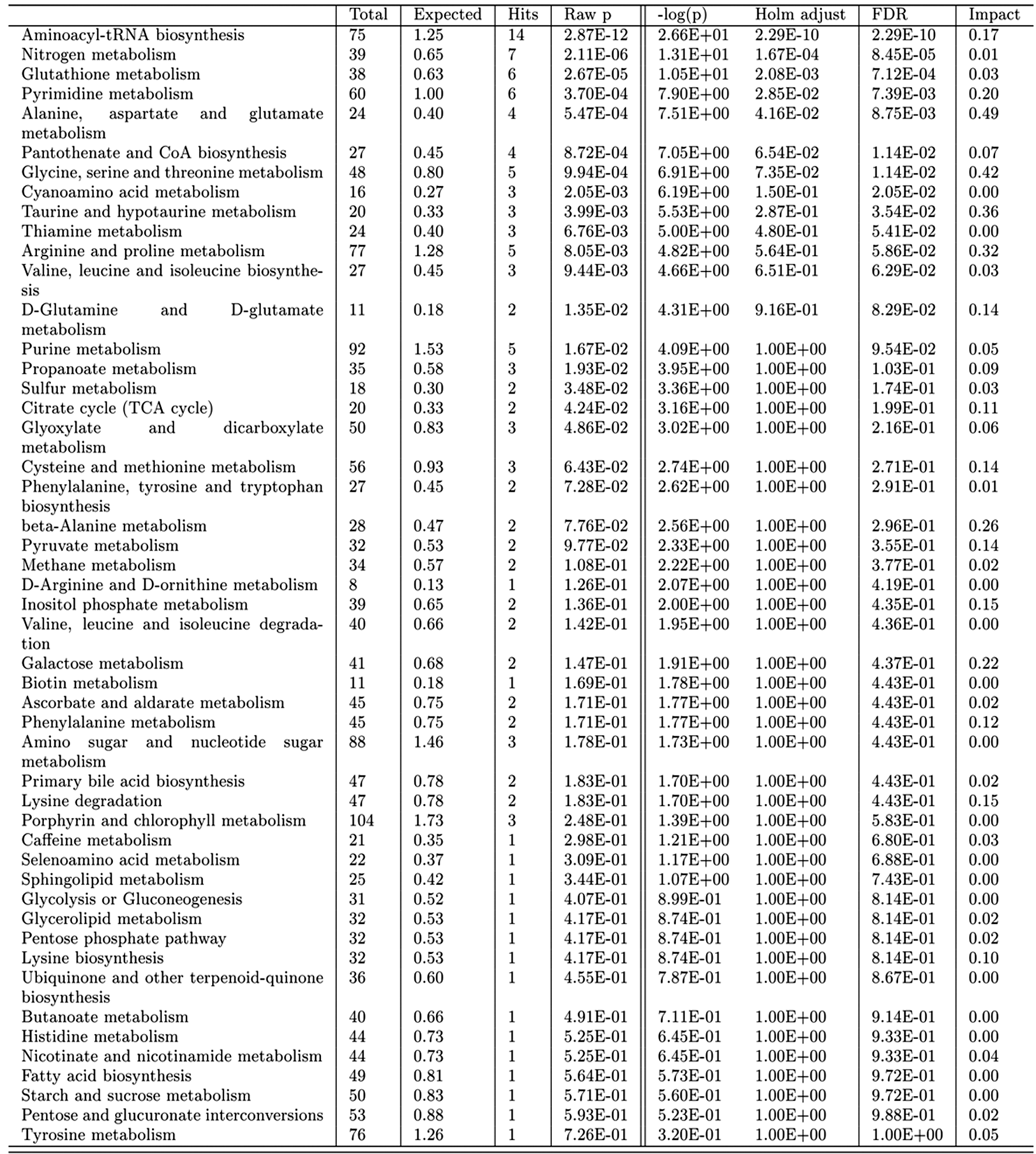


**Table S-4** Metabolic pathway analysis based on the metabolites in culture media for MCF-7S treated with adriamycin


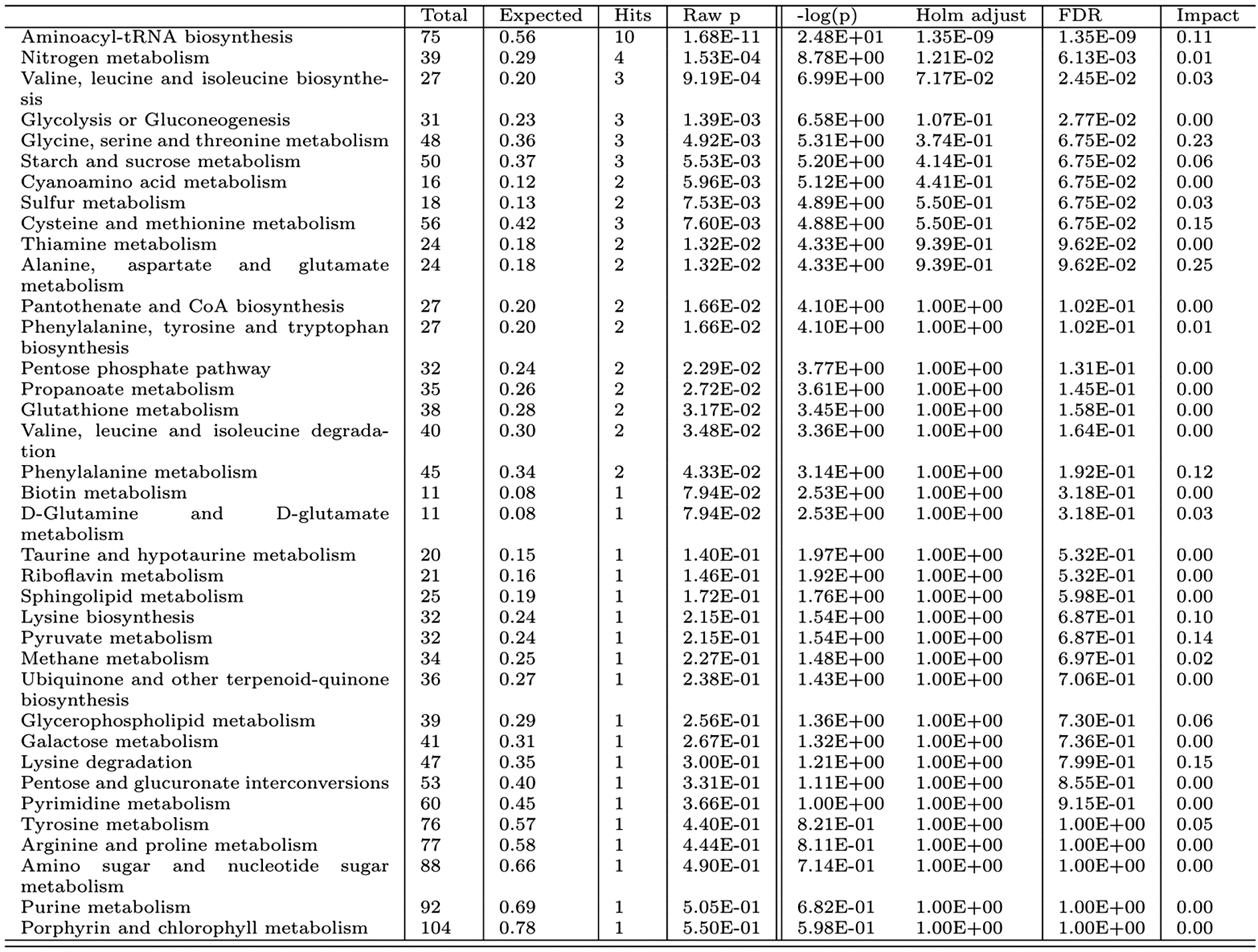


**Table S-5 Distance moved by each group relative to the non-treated control after exposure to adriamycin based on the PLS-DA models ***

| Time  ( h ) | PLS-DA model based on the data of intracellular metabolites | | PLS-DA model based on the data of metabolites in culture media | |
| --- | --- | --- | --- | --- |
|  | MCF-7S | MCF-7Adr | MCF-7S | MCF-7Adr |
| 2 | 1.24 | 2.61 | / | / |
| 6 | 1.59 | 3.14 | 1.19 | 0.97 |
| 12 | / | / | 2.35 | 1.43 |
| 18 | 9.89 | 2.33 | / | / |
| 24 | 17.57 | 1.60 | 5.93 | 1.02 |
| 36 | 21.72 | 2.49 | 8.90 | 1.90 |

*, the relative distant values were calculated based on the specific coordinate parameters available in the mathematic PLD-DA model (See Figure 1B, 1C, 1F, 1G, and reference, Aa J., Shao F., Wang G., et al. Gas chromatography time-of-flight mass spectrometry based metabolomic approach to evaluating toxicity of triptolide. Metabolomics, 2011, 7, 217-225).

**Table S-6** Parameters of the models in Figure 1

| Parameter | Fig.1A | Fig.1B | Fig.1C | Fig.1D | Fig.1E | Fig.1F | Fig.1G | Fig.1H |
| --- | --- | --- | --- | --- | --- | --- | --- | --- |
| Principal components | 3 | 4 | 3 | 3 | 4 | 3 | 2 | 3 |
| R2X | 0.681 | 0.614 | 0.529 | 0.595 | 0.700 | 0.636 | 0.519 | 0.610 |
| R2Y | 0.383 | 0.388 | 0.221 | 0.324 | 0.384 | 0.294 | 0.195 | 0.314 |
| Q2Y | 0.277 | 0.230 | 0.177 | 0.241 | 0.273 | 0.215 | 0.165 | 0.254 |


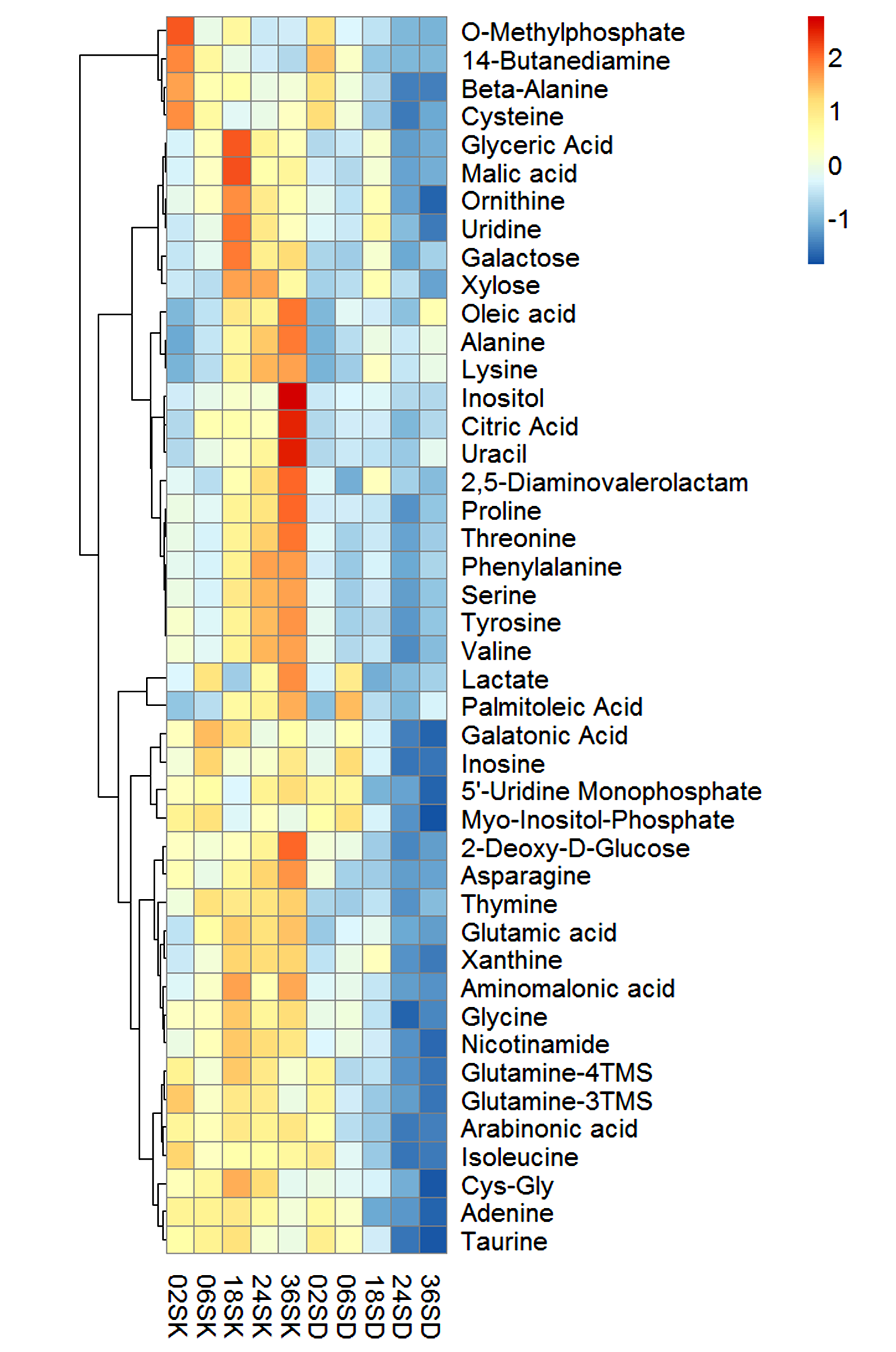


**Figure S-1** Heatmap of the intracellular metabolites for MCF-7S cells in response to adriamycin

**
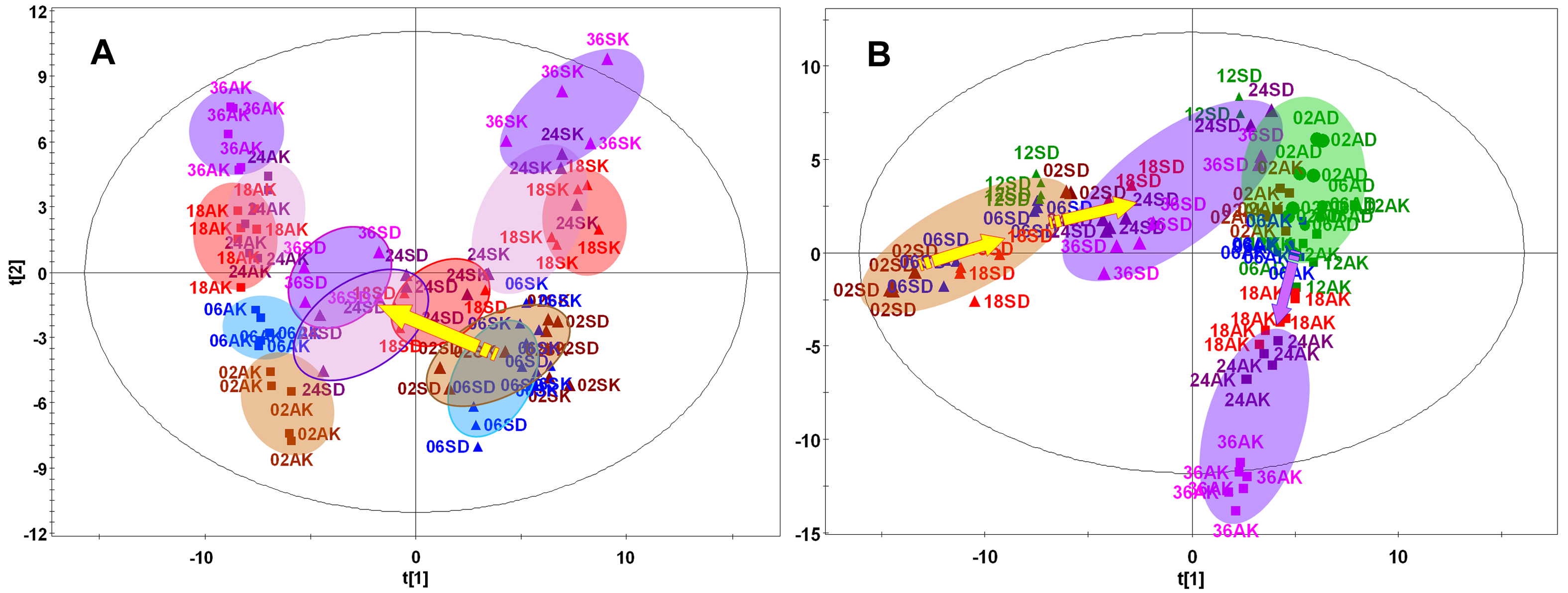
**

**Figure S-2** Metabolic shift of MCF-7S cell intracellular metabolites after adriamycin exposure.


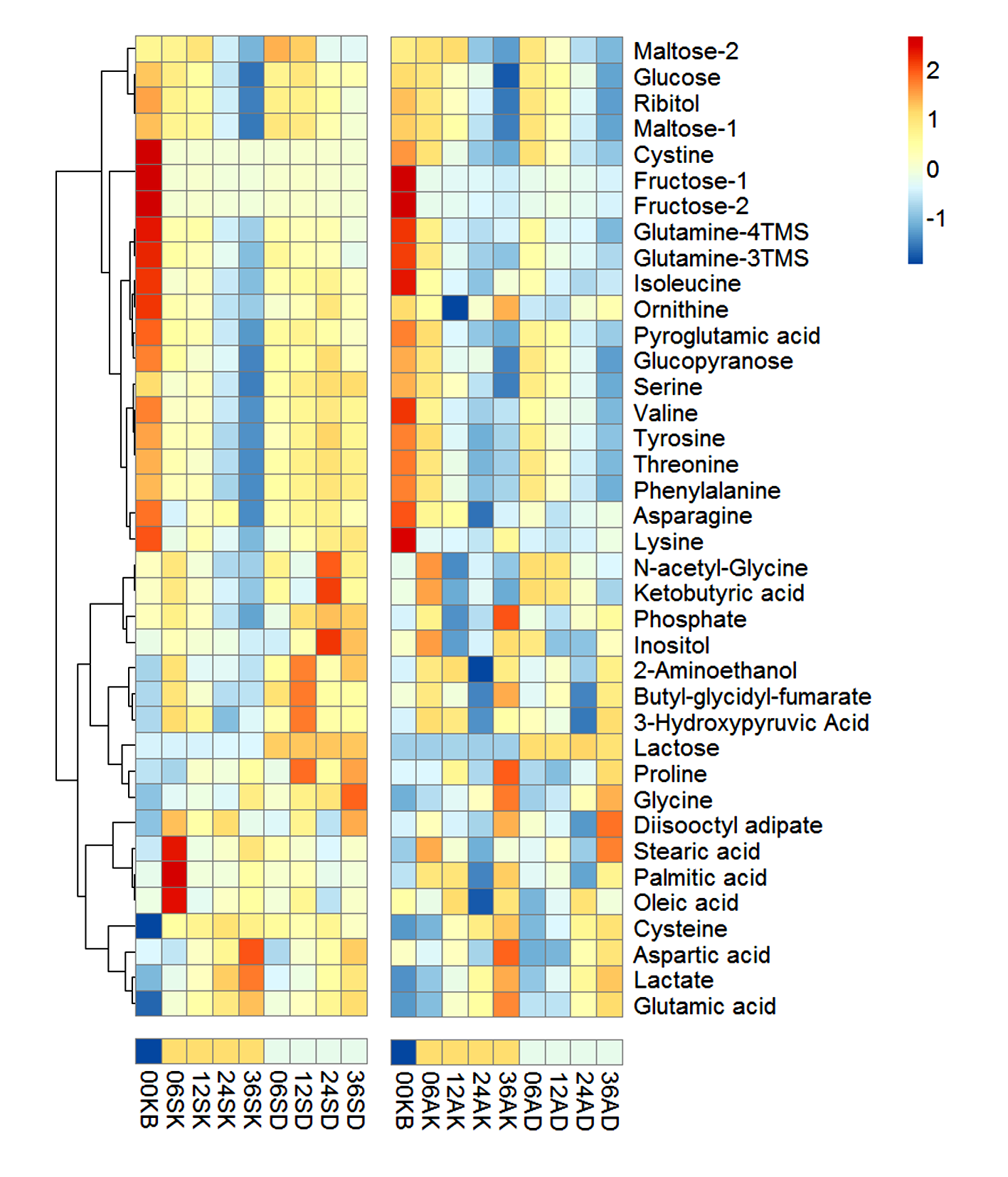


**Figure S-3** Heatmap of the metabolites in the culture media for MCF-7S cells in response to adriamycin


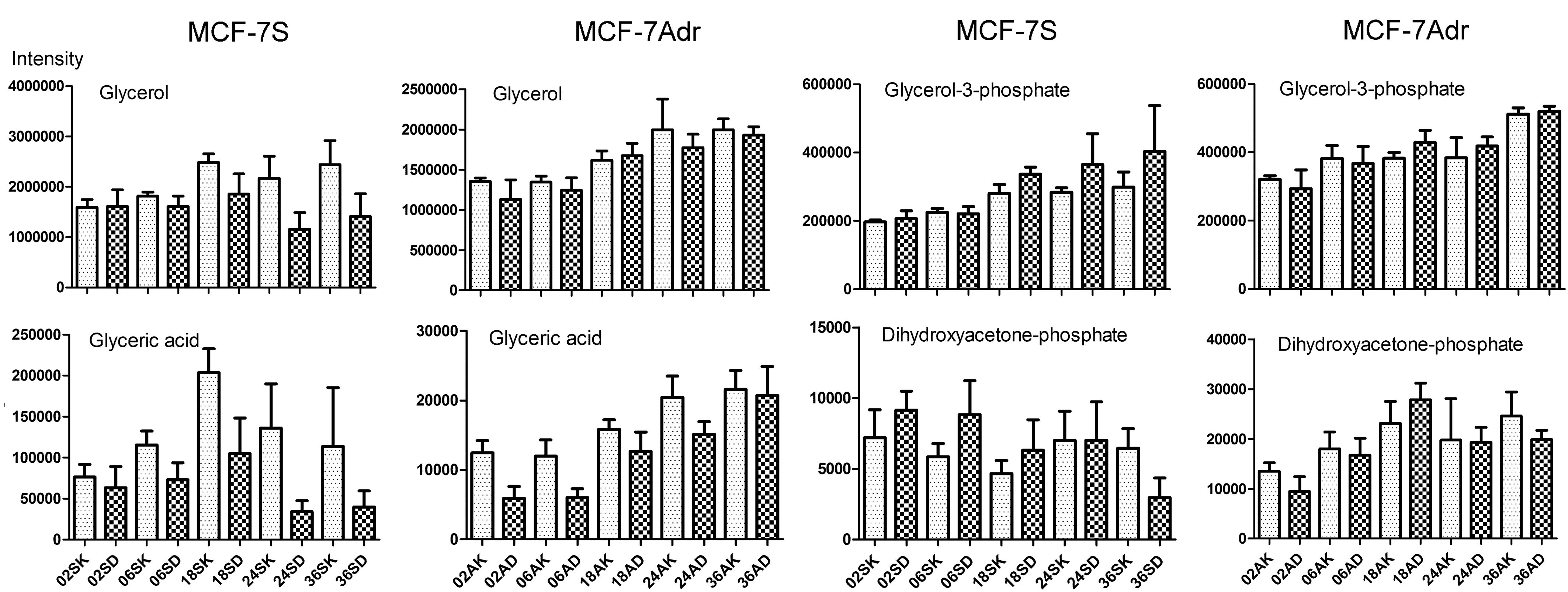


**Figure S-4** The effect of adriamycin on intracellular glycerol metabolism metabolites


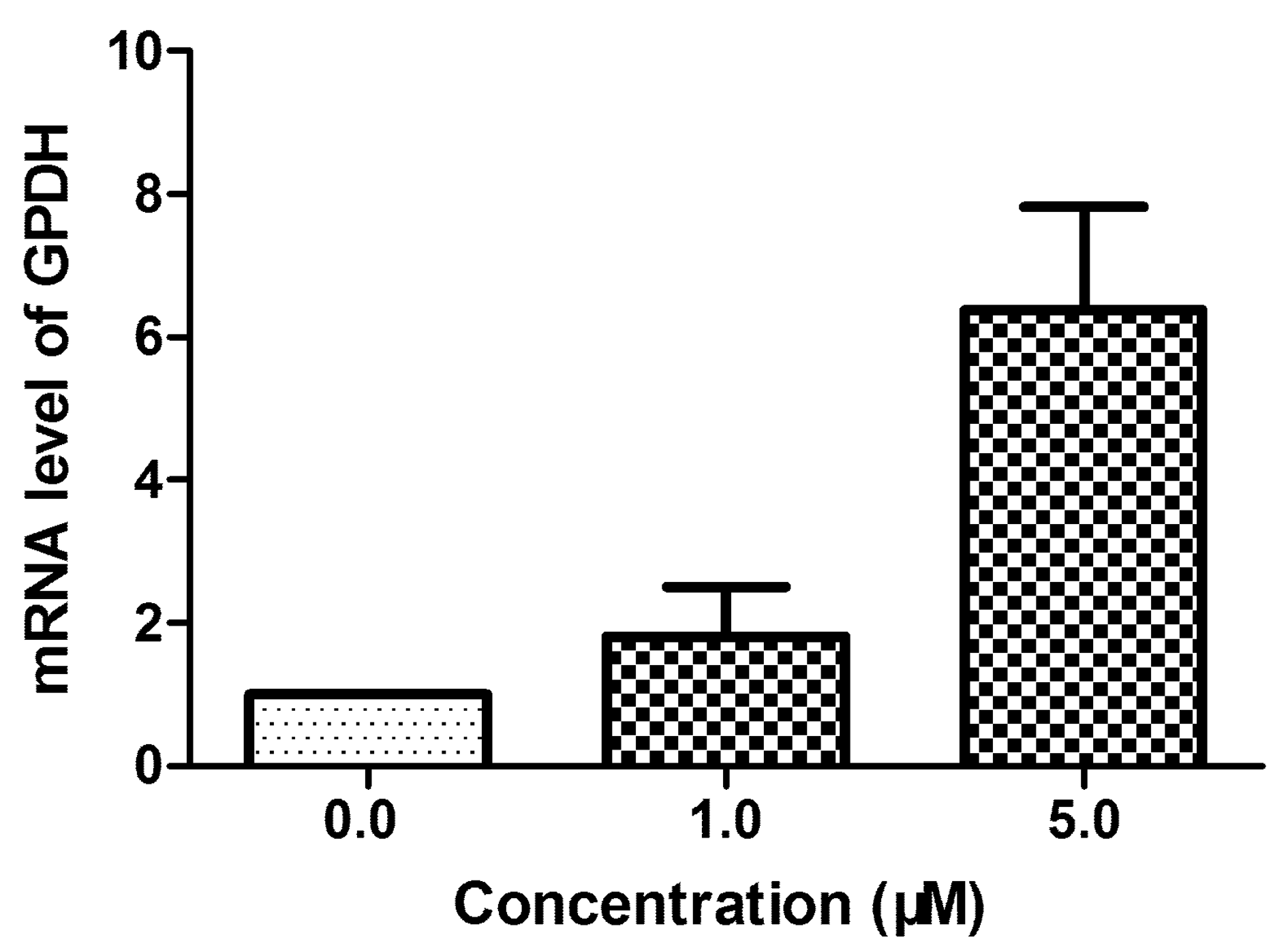


**Figure S-5** mRNA level of GDPH in MCF-7S when exposed to adriamycin for 18 h
